# Flexible CdSe/ZnS Quantum-Dot Light-Emitting Diodes with Higher Efficiency than Rigid Devices

**DOI:** 10.3390/mi13020269

**Published:** 2022-02-07

**Authors:** Mijin Kim, Dongjin Kim, Ohun Kwon, Honyeon Lee

**Affiliations:** Department of Electronic Materials, Devices and Equipment Engineering, Soonchunhyang University, Asan 31538, Korea; jinny3165@naver.com (M.K.); kkddjj4547@naver.com (D.K.); rnjsdhgns159@naver.com (O.K.)

**Keywords:** flexible, quantum-dot light-emitting diode (QLED), oxide/metal/oxide (OMO), MoO_X_/Ag/MoO_X_, SU-8

## Abstract

Fabrication of high-performance, flexible quantum-dot light-emitting diodes (QLEDs) requires the reliable manufacture of a flexible transparent electrode to replace the conventional brittle indium tin oxide (ITO) transparent electrode, along with flexible substrate planarization. We deposited a transparent oxide/metal/oxide (OMO) electrode on a polymer planarization layer and co-optimized both layers. The visible transmittance of the OMO electrode on a polyethylene terephthalate substrate increased markedly. Good electron supply and injection into an electron-transporting layer were achieved using WO_X_/Ag/ WO_X_ and MoOx/Ag/MoO_X_ OMO electrodes. High-performance flexible QLEDs were fabricated from these electrodes; a QLED with a MoO_X_/Ag/ MoO_X_ cathode and an SU-8 planarization layer had a current efficiency of 30.3 cd/A and luminance more than 7 × 10^4^ cd/m^2^. The current efficiency was significantly higher than that of a rigid QLED with an ITO cathode and was higher than current efficiency values obtained from previously reported QLEDs that utilized the same quantum-dot and electron-transporting layer materials as our study.

## 1. Introduction

Flexible electronic displays are in great demand. Most existing flexible displays employ organic light-emitting diodes (OLEDs). Foldable smartphones with OLED displays [1,2,3] are becoming widely available and the first rollable televisions [4] have appeared on the market. Flexible displays widen the applications of electronic displays and greatly reduce manufacturing costs, as they allow for roll-to-roll fabrication [5]. Despite the success of flexible OLEDs, there is a growing demand for flexible devices based on quantum-dot light-emitting diodes (QLEDs). The two types of devices are similar in terms of structure and operation. However, flexible QLED technology is at a very early stage. Recent developments [6,7,8] indicate that flexible QLEDs will perform better than flexible OLEDs, as the environmental vulnerability of organic light-emitting material compromises OLED reliability [9]. QLEDs using inorganic quantum-dot (QD) light-emitting materials can be much more reliable [10]. Moreover, the color purity of QLEDs is much better than that of OLEDs [11] due to QD quantum confinement effects [12,13,14,15]. Therefore, flexible QLED technologies have attracted a great deal of interest.

Flexible QLEDs require a flexible substrate; polyethylene terephthalate (PET) is widely used because of its good transparency in the visible range and high thermal stability. The PET surface is rougher than that of rigid glass; therefore, substrate planarization is required when fabricating high-performance QLEDs on PET substrates [16]. Indium tin oxide (ITO) is widely used as a transparent electrode material for rigid displays given its excellent visible transparency and high electrical conductivity. However, the brittleness of ITO films renders ITO unsuitable for fabrication of flexible transparent electrodes. Dielectric/metal/dielectric (DMD) multilayer flexible transparent electrodes were proposed for replacing ITO transparent electrodes [17]. Several reports on the DMD electrode and surface planarization have appeared [16,17,18,19]; in these studies, their effects were studied separately. However, the effects of DMD electrode fabrication and substrate planarization on device characteristics should be examined at the same time to optimize device performance. In our study, substrate planarization (using a polymer layer) and flexible transparent electrodes (fabricated from a dielectric metal oxide and conducting metal layers) were optimized in combination. Flexible QLEDs of higher efficiency than rigid QLEDs with ITO electrodes were successfully fabricated. Our work will contribute to practical fabrication of flexible QLED devices.

## 2. Experiments

**Figure 1:**
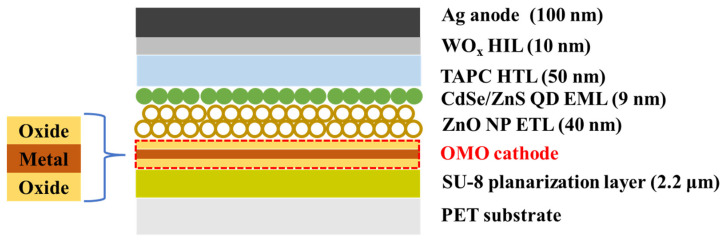
Schematic showing the device structure of flexible QLEDs.

A 180-nm-thick PET film served as the flexible substrate. An epoxy resin (SU-8 2002; Kayaku Advanced Materials) was spin-coated onto the film and annealed at 150 °C for 30 min. A 2.2-μm-thick planarization layer was fabricated on the substrate. As a type of DMD electrode, oxide/metal/oxide (OMO) multilayers served as the flexible transparent electrodes. The dielectric metal oxides WO_X_ and MoO_X_ were used, given their good transparencies. The conducting metal was Ag, given its high electrical conductivity and low visible reflectance [20]. Inverted QLEDs [21] were fabricated using the transparent bottom OMO electrode and planarized substrate, and their properties were examined. The oxide and Ag layers of the OMO electrodes were deposited on the planarization layer via vacuum thermal evaporation at 0.1 nm/s. A ZnO nanoparticle (NP) layer served as the electron-transporting layer (ETL). ZnO NPs were obtained via sol–gel synthesis [22]. A 40-nm-thick ZnO NP ETL was spin-coated onto the OMO electrode using ZnO NPs dispersed in ethanol. QDs with CdSe cores and ZnS shells (CdSe/ZnS) served as the light emitters. CdSe/ZnS QDs with oleic acid ligands (9 nm diameter) were purchased from Global Zeus (Hwaseong, Korea). The photoluminescence (PL) quantum efficiency was >80% and the emission peak PL wavelength and full width at half-maximum were 525 nm and <35 nm, respectively. A QD emission layer (EML) was spin-coated onto the ETL using CdSe/ZnS QDs dispersed in heptane with a 5-mg/mL concentration. The EML was a 9-nm-thick QD monolayer. On the QD EML, a 50-nm-thick di-[4-(*N*,*N*-di-p-tolyl-amino)-phenyl]cyclohexane (TAPC) hole-transporting layer (HTL) was deposited via vacuum thermal evaporation at 0.1 nm/s. On the TAPC HTL, a 10-nm WO_X_ hole-injection layer (HIL) and 100-nm Ag anode were sequentially deposited via vacuum thermal evaporation at 0.1 nm/s. The structure of the fabricated flexible QLEDs is shown in Figure 1.

The current–voltage–luminance (I–V–L) characteristics of the QLEDs were measured in a dark box using an I–V–L tester (Polaronix M6100IVL; McScience Inc., Suwon, Korea) combined with a spectroradiometer (Spectrascan-PR650; Photo Research Inc., Chatsworth, LA, USA). The energy levels of each layer were extracted via ultraviolet photoelectron spectroscopy (UPS) and ultraviolet-visible (UV-Vis) spectroscopy [23]. For UPS analysis, the AXIS Ultra DLD instrument (Kratos Analytical Ltd., Manchester, UK) was used. For UV-Vis spectroscopy, the UV-PC1650 device (Shimadzu Corp., Kyoto, Japan) was employed. Substrate surface morphologies were examined in atomic force microscopy (AFM) images obtained using the XE-7 device (Park Systems Corp., Suwon, Korea).

## 3. Results and Discussion

**Figure 2:**
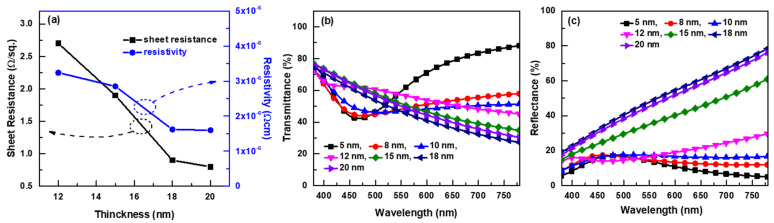
Sheet resistance and resistivity curves of Ag films as a function of film thickness (**a**). Optical transmittance (**b**) and reflectance (**c**) curves in the visible range according to film thickness.

The transmittance and sheet resistance of Ag thin films are important when Ag serves as the metal conductive layer of transparent OMO electrodes. The sheet resistance and resistivity of Ag films as a function of film thickness are shown in Figure 2a. The resistivity decreased as the film thickness increased, and tended to saturate as the thickness increased above 18 nm. For films ≤10 nm thick, the sheet resistance was too high to measure. The optical transmittance and reflectance curves in the visible range are shown in Figure 2b,c according to the film thickness. Films ≤10 nm thick showed plasmonic resonance at a wavelength around 470 nm; thus, the films were a discontinuous network of metallic islands. As the film thickness increased to 12 nm, the transmittance and reflectance curve showed a plateau-like shape; thus, the film formed a continuous percolation network. For the films thicker than 15 nm, the transmittance curves monotonically decreased in visible range; thus, the films were continuous metal films [24]. The reflectance curves of ≥15-nm-thick Ag films were much higher than that of ≤12-nm-thick Ag films. The electrical resistivity values of Figure 2a support the above explanation. The discontinuity of Ag films ≤10 nm thick caused the unmeasurable high sheet resistance; the continuous metal film formation explains the resistivity saturation over 18 nm. The 12-nm Ag film had relatively high transmittance over the entire visible range compared to other films, and its sheet resistance was low enough to be used as a pixel electrode. Considering these advantages, a 12-nm-thick Ag film was chosen as the metal layer of the OMO electrodes.

**Figure 3:**
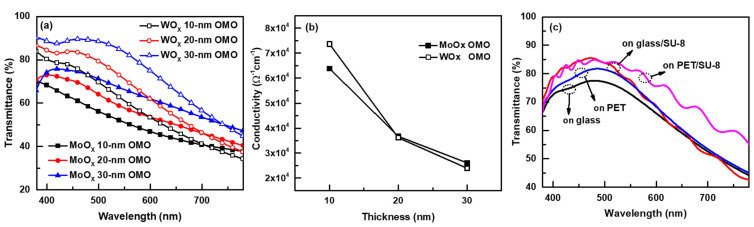
Optical transmittance curves (**a**) and electrical conductivities (**b**) of OMO electrodes according to the oxide layer thickness, and (**c**) optical transmittance curves of 30-nm MoO_X_/12-nm Ag/30-nm MoO_X_ OMO electrodes on different substrates.

PET substrates were coated with a 2.2-μm-thick transparent epoxy resin (SU-8) planarization layer. This reduced the surface roughness from the 0.579-nm Ra of the bare PET film to a 0.225-nm Ra (measured by AFM). The light outcoupling effect of the SU-8 layer was also examined (see below). The properties of OMO electrodes with WO_X_ and MoO_X_ oxide layers were examined. The effects of the oxide layer thicknesses on the optical transmittance of the electrodes were analyzed. The oxide thickness was varied over 10–30 nm with the Ag layer held at 12 nm. The bottom and top oxide layers were of the same thickness; for example, the WO_X_ 10-nm OMO structure was 10-nm WO_X_/12-nm Ag/10-nm WO_X_. Figure 3a shows that the optical transmittance increased according to the oxide layer thickness increase. The reflectance can be reduced by adjusting the phase differences between each reflection wave (reflected at the surface of front oxide, at the Ag metal front surface, and at the Ag metal back surface). The phase difference depends on the refractive index and thickness of oxide layers [20]. This makes the optical transmittance depend on the oxide thickness. The oxide thickness was limited to 30 nm, as a thicker oxide layer makes charge injection from the Ag layer into the ZnO NP ETL difficult. The electrical conductivity of the Ag layer decreased as the oxide layer thickness increased (Figure 3b). Oxygen diffusion into the Ag layer (from the oxide layers) may increase as oxide thickness increases; this may partly oxidize the Ag and thus reduce conductivity. The effect of SU-8 planarization on the visible transmittance of the OMO electrodes was examined; the transmittance curves of the MoO_X_ 30-nm OMOs on bare glass, SU-8-coated glass, bare PET, and SU-8-coated PET are shown in Figure 3c. The references were the bare substrates; hence, the curves are OMO transmittance curves of bare substrates or OMO/SU-8 transmittance curves of SU-8 coated substrates. The transmittance was higher for the latter materials, particularly for the PET substrate. The thick dielectric layer of SU-8 may extract light trapped in surface plasmon polariton (SPP) modes at the surface of the Ag metal layer [25], thus increasing optical transmittance.

**Figure 4:**
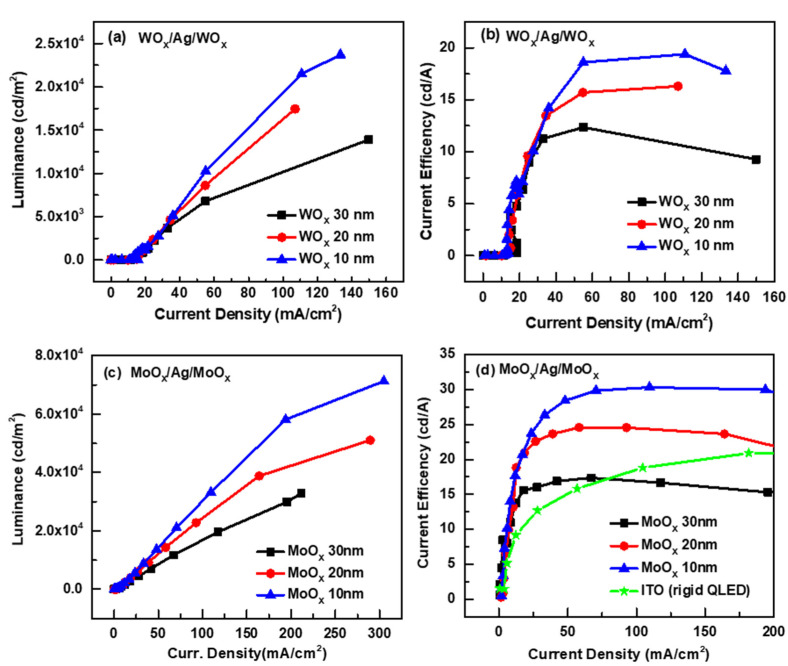
Luminance (**a**) and current efficiency (**b**) curves of QLEDs with WO_X_/Ag/WO_X_ OMOs, and luminance (**c**) and current efficiency (**d**) curves of QLEDs with MoO_X_/Ag/MoO_X_ OMOs. The current efficiency curve of the rigid QLED marked “ITO (rigid QLED)” is shown in Figure 4d.

The device characteristics of flexible QLEDs with OMO cathodes are shown in Figure 4 (OMO with WO_X_, Figure 4a,b; OMO with MoO_X_, Figure 4c,d). Maximum current efficiency values according to the oxide thickness of OMO cathodes are summarized in Table 1. OMOs with 10-nm-thick oxide layers performed best. Device performance decreased as oxide thickness increased, despite an increase in optical transmittance (Figure 3). Electron injection from the Ag layer into the ZnO ETL became more difficult as the oxide layer became thicker due to its high electrical resistivity. Thus, the electron injection properties affected device performance more so than the optical transmittance. Performance depended mainly on oxide layer thickness. Performance can be improved by optimizing both the electron injection efficiency and optical transmittance. The current efficiencies of QLEDs with WO_X_ and MoO_X_ oxide layers were 19.4 and 30.3 cd/A, respectively. These are equal to or larger than the values of previous reports using QLEDs with CdSe/ZnS QD EMLs and ZnO ETLs [26,27,28,29]. The EML and ETL materials greatly affect device performance; we therefore evaluated the effects of the transparent bottom cathode and substrate planarization layer on performance compared to those of QLEDs fabricated from the same EML and ETL materials employed in our study.

**Figure 5:**
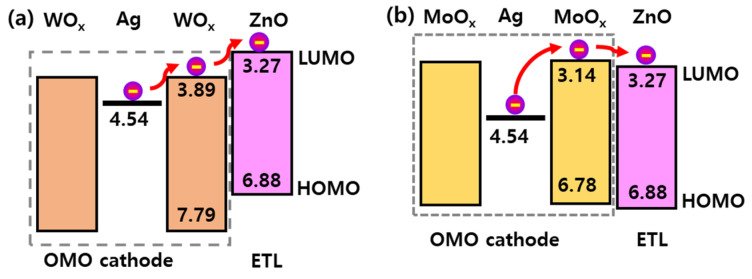
The energy levels of (**a**) the WO_X_/Ag/WO_X_ OMO and ZnO NP ETLs and (**b**) MoO_X_/Ag/MoO_X_ OMO and ZnO NP ETLs.

QLEDs with MoO_X_ performed better than QLEDs with WO_X_. This can be explained by the energy level alignment (Figure 5). The energy levels of each layer were extracted by UPS and UV-Vis analyses. The lowest unoccupied molecular orbital (LUMO) level of WO_X_ was approximately equidistant from the Fermi level of the Ag layer and the LUMO level of the ZnO ETL. The MoO_X_ LUMO level was above the ZnO ETL LUMO level. Thus, electron injection from the Ag layer into the ZnO ETL is dominated by thermionic emission in the case of WO_X_ and tunneling in the case of MoO_X_ [30]. For high resistivity thin films such as WO_X_ and MoO_X_, tunneling is better to transport electrons than thermionic emission; this explains the better performance of QLEDs with MoO_X_. The flexible QLED with MoO_X_ exhibited a noticeably higher efficiency (30.3 cd/A) than the rigid QLED (Figure 4d) fabricated on a glass substrate, which was identical to the flexible QLED except that an ITO cathode replaced the OMO cathode and there was no planarization layer.

## 4. Conclusions

We used flexible transparent OMO electrodes and flexible substrate (polymer) planarization to fabricate flexible QLEDs with efficiencies exceeding those of rigid QLEDs. The current efficiency of the flexible QLED with an MoO_X_/Ag/MoO_X_ cathode and SU-8 planarization layer was 30.3 cd/A, which was significantly higher than that of a rigid QLED with an ITO cathode, and significantly higher than that of previous QLEDs using the same QD and ETL materials as those used in our study. This was because we simultaneously optimized the optical transmittance of the OMO cathode and electron injection from the OMO cathode to the ETL. However, further work is needed prior to extensive commercialization of flexible QLEDs. It is important to further optimize both the optical transmittance and electron injection capability of the OMO electrodes. Additionally, there is a need to improve OMO/SU-8 optical transmittance. Moreover, a reliable encapsulation optimized for flexible QLEDs is required to achieve a practical level of stability [31].

## Figures and Tables

**Table 1:** Current efficiency values according to OMO oxide thickness.

Oxide Thickness (nm)	Maximum Current Efficiency (cd/A)	
	WO_X_/Ag/WO_X_	MoO_X_/Ag/MoO_X_
10	19.4	30.3
20	16.3	24.6
30	12.3	17.4

## Data Availability

Not applicable.
